# Optimism Bias in Fans and Sports Reporters

**DOI:** 10.1371/journal.pone.0137685

**Published:** 2015-09-09

**Authors:** Bradley C. Love, Łukasz Kopeć, Olivia Guest

**Affiliations:** 1 Experimental Psychology, University College London, London, United Kingdom; 2 Experimental Psychology, University of Oxford, Oxford, United Kingdom; University of California, San Diego, UNITED STATES

## Abstract

People are optimistic about their prospects relative to others. However, existing studies can be difficult to interpret because outcomes are not zero-sum. For example, one person avoiding cancer does not necessitate that another person develops cancer. Ideally, optimism bias would be evaluated within a closed formal system to establish with certainty the extent of the bias and the associated environmental factors, such that optimism bias is demonstrated when a population is internally inconsistent. Accordingly, we asked NFL fans to predict how many games teams they liked and disliked would win in the 2015 season. Fans, like ESPN reporters assigned to cover a team, were overly optimistic about their team’s prospects. The opposite pattern was found for teams that fans disliked. Optimism may flourish because year-to-year team results are marked by auto-correlation and regression to the group mean (i.e., good teams stay good, but bad teams improve).

## Introduction

Optimism bias refers to people's tendency to overestimate the probability of experiencing positive outcomes and underestimate the probability of experiencing negative outcomes [1, 2]. For example, people have overly rosy forecasts of their prospects in regards to traffic accidents [3], cancer risk [4], and work-place safety [5]. Optimism bias may be so prevalent because it is associated with improved health outcomes [6] and workplace performance [7], whereas realistic expectations are associated with depression [8]. On the other hand, optimism bias can have negative consequences, such as not seeking needed medical care because of underestimated risk [9].

Unfortunately, interpreting results in real-world domains can be non-trivial to the point where some question whether there is an optimism bias [10]. For example, when people are optimistic about their cancer prospects, perhaps they are accessing additional information about their family history or believe medical science will advance in the coming years. Testing for optimism bias involves relating an environmental statistic (e.g., cancer rate) to the participant sample in the study and this sample may not be drawn from the same distribution that generated the statistic. Skewed distributions can also create issues when people fail to appreciate the difference between the mean and the median. For example, almost every human has more arms and legs than the average. Furthermore, what it means to be “better” than average is malleable, multidimensional, and open to interpretation in many cases. For example, there are numerous ways people could construe being a better than average driver. At its heart, optimism bias involves believing that one will fare better than average. However, in all these examples, one’s own outcome is not zero sum with those of others. One person avoiding cancer does not necessitate that another person develops cancer. One research challenge is conclusively evaluating whether optimism bias exists.

Ideally, optimism bias would be evaluated within a closed formal system to establish with certainty when the bias occurs, the extent of it, and the associated environmental predictors. In such a system with zero-sum outcomes, optimism bias is conclusively shown when people’s predictions are collectively inconsistent. In this report, we consider one such formal system: the National Football League (NFL). When two teams play, only one team can go home victorious. Therefore, if fans forecast more wins than average across teams, they are collectively biased.

We test whether optimism bias extends to people’s predictions for their favorite team. We also ask the related question of whether fans predict negative outcomes for the team they most dislike. Although a team’s success is not the same as a fan’s personal success, people have close allegiance to teams they support. We test for this two-fold pattern of optimism bias in fandom–inflated predictions for people’s most beloved team and lower predictions for a despised rival. By aggregating across fans, we can evaluate whether people are inconsistent in their predictions.

This fandom form of optimism bias may extend to experts, who in other domains are susceptible to optimism bias [11] and may be no better at forecasting than novices [12]. Experts assigned to cover a team may become self-interested in the result and biased. As experts learn more about a particular team, they may only focus on improvements and changes to their team and not fully appreciate the changes made at other teams (cf. [13]). Attention may become biased toward positive attributes for a team that is closely followed (cf. [14, 15]). An article published by ESPN prior to the start of the 2014 NFL season provides a natural experiment [16]. An expert was assigned to each of the 32 NFL teams and predicted the final record for their team. Conclusively demonstrating the optimism bias, the mean prediction was 8.93 wins for the 16 game season, which is significantly greater than 8, the average number of wins possible, *t*(31) = 2.67, **p** ≈.01. Parenthetically, experts’ forecasts were no better correlated with the observed wins (r = .62) than a naive forecasting model that predicted that each team would duplicate its 2013 win total in 2014 (r = .59).

One advantage of considering prediction within a formal system, such as the NFL, is that hypotheses about how the statistical structure of the environment shape decision making can be evaluated (cf. [17]). Historic patterns of team performance may provide insight into why fans remain optimistic in the face of perennial losses. Examining the last ten NFL seasons, a good predictor of whether a team will improve on the previous season is how many games the team won the previous season, **z** = -7.76, **p≈**0. The following logistic regression solution describes the relationship:

$$P\left(improve\right)=\frac{1}{1+e^{-\left(2.87-0.39x\right)}}\;,$$ (1)

where *x* is the number of games won the previous season. In this sense, one is correct to be optimistic about the future prospects of a moribund team as improvement is likely. According to Eq 1, a team that won 4 games the previous season has a 79% chance of improving this season, whereas a previous 12-game winner only has a 14% chance. Thus, optimism for poor teams is validated by improvement year on end. At the same time, performance from one season to another is auto-correlated, *t*(318) = 6.10, **p**≈0, a typical quality of real-world environments [18]. Predicting wins for a season from the win total from the previous season (analyzing the last ten seasons), yields the regression solution

$$x_{next}=0.32x+5.40\;,$$ (2)

indicating that a team that wins 4 games one season can be expected to win 6.70 the next season, whereas a team with 12 wins the previous season can expect 9.30 wins. Thus, poor teams should improve, but strong teams should still be above average from season to season. This pattern of results, featuring regression to the group mean and auto-correlation, would seem to allow most fans to find a silver lining, perhaps not piercing their veil of optimism. If one is motivated to retain a positive outlook, certain aspects of this environment can be selectively attended.

## Study

Our prediction is that the NFL environment structure will be supportive of optimism bias. Fans, like ESPN experts, should treat the outcome of their team as akin to their own prospects and be optimistic. This optimism should also extend to predicting negative outcomes for rivals. A large-scale study of NFL fans tested these predictions.

### Method

#### Participants

UCL Experimental Psychology ethics committee approved this research. US-based NFL fans were recruited using Amazon Mechanical Turk, a paid online crowdsourcing platform, which is an effective method for recruiting demographically diverse samples [19] and has been shown to yield results consistent with decision-making studies in the laboratory [20]. The study was run for three days (April 17–19, 2015) during the NFL offseason with the target of collecting as much data as possible within a budget allowing for a maximum n of 2000. Over those three days, 1118 participants completed the very brief study for $0.25 compensation. Two participants were excluded for choosing the same team as both their most and least favorite. Of the remaining 1116 participants, the mean age was 34.46 years (sd = 10.84) with 446 females and 670 males.

#### Procedure and Design

Using dropdown menus, participants indicated their favorite team (listed in alphabetical order). A second dropdown menu (0–16) was used to predict the win total for the 2015 season. The same procedure was used for to assess predictions for participants’ least favorite team with the order (most liked vs. least liked) counterbalanced across participants. To assess participants’ knowledge of the NFL, participants were asked how many yards the penalty was for offsides, choosing between the options of 0, 5, 10, and 15 yards. The vast majority (86.11%) of participants correctly answered 5 yards.

### Results

**Fig 1:**
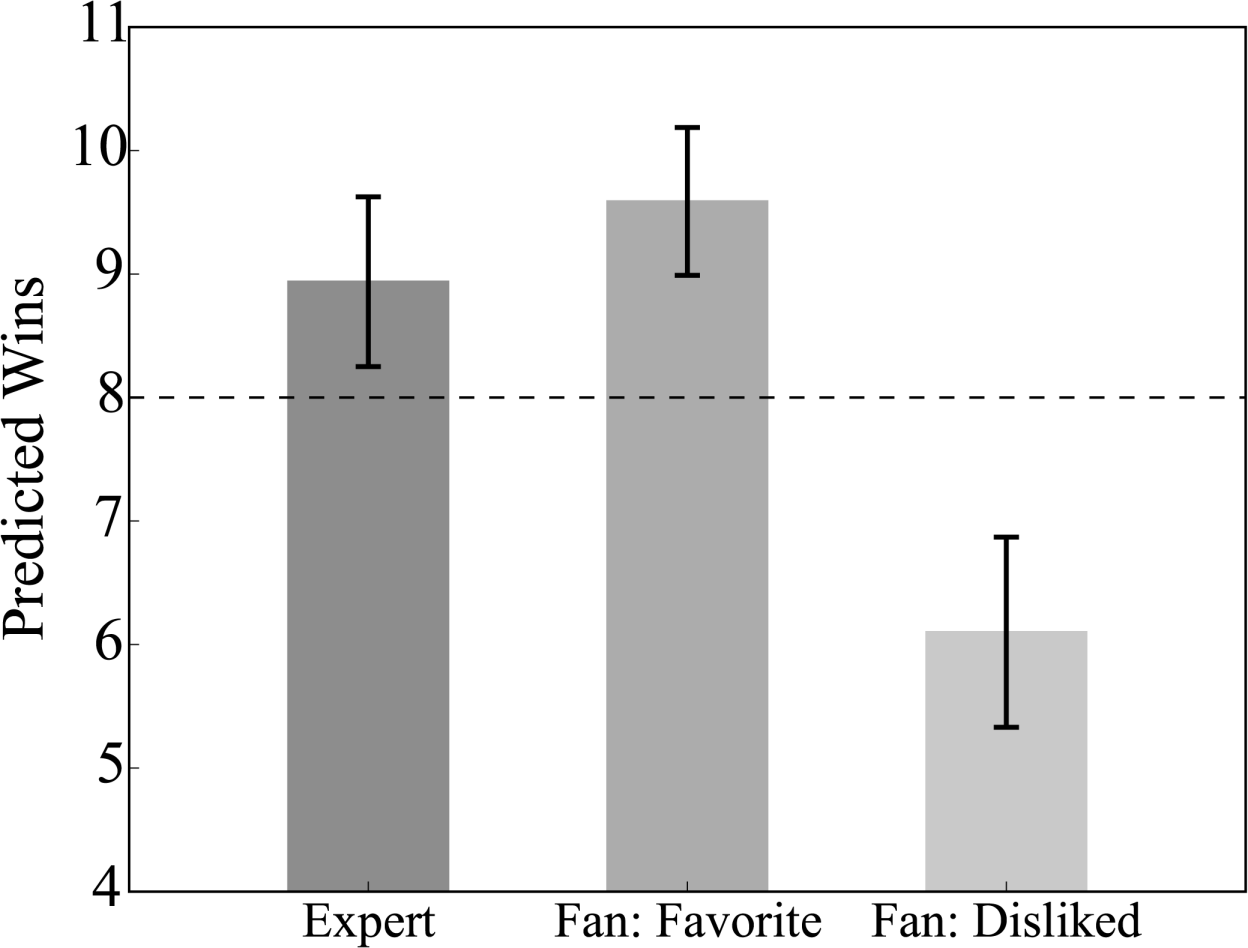
Wins over teams must average to 8 games in a 16 game season. In contrast, ESPN experts collectively overestimate the number of wins for teams they follow, as do fans for their favorite team. In contrast, fans underestimate wins for teams they dislike. Error bars are 95% between-subject confidence intervals.

The main dependent measure was the average win projection for each team averaged across participants. Notice that this analysis by team is not biased to overweight successful teams, which may be more popular with fans. The main results are shown in Fig 1. NFL fans predicted more wins for teams they favored (9.59) than teams they disliked (6.10), *t*(31) = 12.53, **p**≈0. Both of these estimates are significantly different from the logically necessary mean of 8, *t*(31) = 5.19, **p**≈0, *t*(31) = -4.83, **p**≈0, respectively. When only the participants who correctly answered the offsides question are considered, the difference between favored and disliked teams increases (10.30 vs. 6.10).

An additional analysis was conducted in which responses were not aggregated by team. Instead, team was included as a factor to capture variance associated with the differing win expectations across teams. An ANOVA with judgment type (i.e., liked vs. disliked), team, and subject included as factors found a significant effect for expected wins depending on whether fans were rating a team they liked or disliked, F(1,1084) = 771.62, **p**≈0. These results strongly support the primary hypothesis–optimism bias extends to fandom such that people overestimate wins for their favorite team and underestimate wins for their most disliked team.

In Introduction, we stressed that the auto-correlative nature of the environment may help induce the optimism bias (see Eq 2). People’s predictions for the 2016 season were correlated with the number of wins for the 2015 season for favored and disliked teams, *t*(30) = 9.67, **p**≈0, *t*(30) = 5.11, **p**≈0, respectively. The regression equation for favored teams is

$$x_{next}=0.47x+5.80\;,$$ (3)

and for disliked teams is

$$x_{next}=0.48x+2.28\;.$$ (4)

Notice that Eqs 3 and 4 differ primarily in intercept and match in slope (i.e., the auto-correlative term). Both slopes are steeper than the environmental estimate shown in Eq 2. Thus, Eqs 3 and 4 indicate that people are sensitive to the environment and perhaps believe it is more auto-correlated across seasons than it actually is.

As a point of curiosity and to further confirm that our participants were NFL fans, we examined which teams were most liked, disliked, and appeared in rivalries (see Table 1). The most liked teams have been recently and historically successful. Notice that many of the same teams that are most liked are also most disliked. Across all 32 teams, the correlation between the frequency of being listed as liked and disliked was r = .62, *t*(30) = 4.33, **p**≈0. Although it is possible that participants chose teams based on expected performance, this result, along with the others, suggests that participants were actual fans. One interesting observation is that more people listed the New England Patriots as their most disliked team than the sum of the 14 least frequently listed disliked teams.

**Table 1:** Teams most liked and disliked by fans. The most popular rivalries (i.e., combinations of like and dislike) are also shown. The proportion of total responses is shown in parentheses. The final two columns show the largest and smallest difference in expected wins between those who like and dislike a team.

Most Liked	Most Disliked	Most Popular Rivalries	Largest Optimism Gap	Smallest Optimism Gap
New England Patriots (7.7%)	New England Patriots (17.3%)	Dallas Cowboys and Philadelphia Eagles (1.7%)	Cincinnati Bengals (6.6 games)	Denver Broncos (0.7 games)
Green Bay Packers (7.6%)	Dallas Cowboys (13.7%)	Chicago Bears and Green Bay Packers (1.7%)	Arizona Cardinals (6.6 games)	Seattle Seahawks (1.0 games)
Dallas Cowboys (6.5%)	Oakland Raiders (6.0%)	New York Giants and New England Patriots (1.5%)	Houston Texans (6.4 games)	New England Patriots (1.0 games)
New York Giants (6.0%)	Pittsburgh Steelers (4.7%)	New York Jets and New England Patriots (1.3%)	Kansas City Chiefs (5.6 games)	Philadelphia Eagles (1.1 games)
Pittsburgh Steelers and Seattle Seahawks (tie, 5.7%)	Green Bay Packers (4.5%)	Dallas Cowboys and Washington Redskins (1.1%)	Carolina Panthers (5.6 games)	Pittsburgh Steelers (2.1 games)

The top rivalries should also be familiar to NFL fans. Many of these rivalries are long-standing, whereas others reflect recent Super Bowl matchups. Notice that even the most popular rivalries are not that frequent in absolute terms. Overall, there was a non-zero entry for 313 of the 496 (32×31/2) possible rivalries.

Table 1 also shows largest and smallest differences in expected wins between those who like and dislike a team. Notice that the teams with the smallest differential have been successful recently and receive a great deal of national media attention, whereas teams with the largest differential tend to be middle of the road.

## Discussion

Fans, like professionals assigned to cover a team, were overly optimistic about their team’s prospects. The opposite pattern was found for teams that fans disliked. Because success within the NFL is zero sum, these results make clear that bias exists and that collective decision making is inconsistent. Our analyses cast optimism bias as a property of a population rather than an individual.

Conducting studies within a domain that has objective outcomes and provides rich information about the environmental structure has a number of advantages. One can entertain hypotheses like that optimism bias will flourish in environments that are auto-correlated and that show regression toward a group mean. In the case of the NFL, we speculated that fans in that environment could always find a reason to be somewhat hopeful about their team’s prospects. This explanation is consistent with the related idea that people’s attention is somewhat motivated and that fans may focus on the positive aspects of their favorite team (cf. [14, 15]). Perhaps consistent, notice that the optimism gap (see Table 1) is largest for middle of the road teams that receive little national media coverage, enabling fans and local media to construct their own narratives. These conjectures await further testing. We hope the ease of measuring and manipulating such information proves fruitful in future research.

We find that optimism bias is not limited to one’s own prospects, but extends to those dear to an individual, such as a favored team. These results align with the finding that people judge their friends to be better than average [21, 22]. Perhaps these positive beliefs about close associates confer many of the proposed advantages of the optimism bias to others. Indeed, these positive evaluations of close associates may contribute to recipients developing the optimism bias. In effect, positivity may be contagious even in situations where there are only so many winners.
